# The Physiological Value of Beer

**Published:** 1911-09-30

**Authors:** 


### The Hospital

A JOURNAL OF

The Medical Sciences and Hospital Administration.

NEW SERIES. No. 244, VOL. IX. [No. 1316, VOL. L.] SATURDAY, SEPTEMBER 30, 1911.

### THE PHYSIOLOGICAL VALUE OF BEER.

Two years ago an International Dietetic Physiological Research Institute, one of whose objects was research into the physiological and dietetic properties of beer, was founded in Berlin. Subscriptions were obtained towards the expenses of the Institute from the Institute of Brewing and the Brewers' Society in this country, and from similar associations in other beer-producing countries. Since its inception, this Institute has done some pioneering work in research connected with dietetics, especially with reference to the action of alcohol upon the products of digestion and the process of digestion itself. Amongst other questions of distinct medical complexion which have been taken in hand, some researches on diabetes and the use of yeast preparations in its treatment may be mentioned. From the more strictly dietetic and physiological standpoint, however, a research lately published on the physiological value of beer in man and lower animals is probably more important. The investigation has been conducted throughout on the most approved scientific lines, and the conclusions reached would appear to be fairly warranted by the facts and figures collected in support of them. The most important of these conclusions, which with the experimental work are set forth in full in Pflüger's "Archives of Physiology," are as follows:—

Beer extract, added to a dietary almost devoid of organic condiments, increases the assimilation of the non-nitrogenous foodstuffs, particularly of fats, and is therefore to be regarded as a condiment the value of which can be expressed in definite numerical terms. It is, of course, well known that salt, mustard, pepper, peppermint, and alcohol have this same property of assisting in the assimilation of food; and if the extract of beer also possesses it, clearly beer has a food value quite apart from any alcohol it may contain. The nitrogenous content of beer is so small that, although 40 per cent. of it is assimilable, in any case its food value as far as nitrogen is concerned is very low.

The next point is the effect on the beer extractives of the alcohol which is associated with them in commercial beer. As a fact, the inquiry shows that whereas of beer extract as such added to normal diet about 86 per cent. is assimilated, only 80 per cent. is assimilated when the beer extract is administered as part of a drink of ordinary beer. In other words, the associated alcohol reduces the assimilation of beer extractives by 6 per cent., although it reduces that of the associated foodstuffs by less than 1 per cent. These figures were obtained by feeding experiments on animals. When the experiments were repeated on man, similar results in the main were obtained. It appeared, however, that in man the extractives are more completely utilised than by dogs, and in one case the physiological efficiency of beer amounted to 91.2 per cent. of its thermal value.

These experiments entirely support the conclusions arrived at by the body of experts who carried out the important investigations into the dietetic value of beer on behalf of THE HOSPITAL. A summary of these results, which at the time attracted considerable comment and some criticism, was published in the special report of THE HOSPITAL Commission on Beer (THE HOSPITAL, April 24, 1909). Our commissioners found that the dietetic value of beer was considerable, and they carefully investigated the relation between thermal and dietetic value, with the result that they obtained certain data closely corresponding to those now furnished by the International Dietetic Institute. Since then we have been favoured with the findings of independent investigators whose results appear to be analogous.

These data do not prove, and no one would wish to contend otherwise, that beer is a necessary ingredient of diet. But they do go far to show that when taken in moderation beer has a very marked dietetic value, and that when excess is duly avoided nothing but benefit can follow its ingestion by normal healthy individuals. When we take into consideration the fact that the better-class English beers, as manufactured nowadays, are almost ideal thirst-quenching beverages, and that, everything taken into account, no purer commercial product is obtainable than the better brands of beer emanating from the factories of our reputable brewing companies, there is some justification for asserting that the national drink ought to claim a greater indulgence from the medical profession than it has hitherto obtained. Its dietetic good qualities are indisputable, and its food value is definite. This does not in any way affect the further question whether, since many who partake of beer have not sufficient stability to practise moderation, those who can do so ought or ought not to abstain totally from it for the sake of example. That is a quite different question, and one which it is no part of our purpose to discuss here.

